# Single-molecule mechanostructural fingerprinting of nucleic acid conformations

**DOI:** 10.1093/nar/gkaf1465

**Published:** 2026-01-06

**Authors:** Prakash Shrestha, Hans T Bergal, William M Shih, Wesley P Wong

**Affiliations:** Department of Chemistry, University of Kentucky, Lexington, KY 40506, USA; Program in Cellular and Molecular Medicine, Boston Children’s Hospital, Boston, MA 02115, USA; Wyss Institute for Biologically Inspired Engineering, Harvard University, Boston, MA 02115, USA; Department of Biological Chemistry and Molecular Pharmacology, Blavatnik Institute, Harvard Medical School, Boston MA 02115, USA; Program in Cellular and Molecular Medicine, Boston Children’s Hospital, Boston, MA 02115, USA; Department of Biological Chemistry and Molecular Pharmacology, Blavatnik Institute, Harvard Medical School, Boston MA 02115, USA; Wyss Institute for Biologically Inspired Engineering, Harvard University, Boston, MA 02115, USA; Department of Biological Chemistry and Molecular Pharmacology, Blavatnik Institute, Harvard Medical School, Boston MA 02115, USA; Department of Cancer Biology, Dana–Farber Cancer Institute, Boston, MA 02115, USA; Program in Cellular and Molecular Medicine, Boston Children’s Hospital, Boston, MA 02115, USA; Wyss Institute for Biologically Inspired Engineering, Harvard University, Boston, MA 02115, USA; Department of Biological Chemistry and Molecular Pharmacology, Blavatnik Institute, Harvard Medical School, Boston MA 02115, USA; Department of Pediatrics, Harvard Medical School, Boston, MA 02115, USA

## Abstract

Understanding the three-dimensional structure and mechanical response of biomolecules is key to uncovering their molecular mechanisms, particularly in contexts where force plays a regulatory role. Structural methods such as X-ray crystallography, Cryo-electron microscopy, and Nuclear Magnetic Resonance (NMR) spectroscopy provide high-resolution conformational data, while single-molecule force spectroscopy reveals mechanical properties—but these approaches are rarely integrated. A more comprehensive understanding of structure-function relationships, including nonequilibrium conformations and transitions under force, calls for methods capable of simultaneously resolving structural and mechanical properties at the single-molecule level. To meet this need, we present a DNA nanoswitch calipers platform capable of both measuring multiple intramolecular distances and mechanically unfolding individual biomolecules along defined axes. Using human telomeric DNA G-quadruplexes as a model system, we mapped distances between labeled sites to distinguish conformational states and performed directional unfolding to characterize mechanical stability along defined axes. This integrative approach revealed subtle conformational and mechanical differences, showcasing DNA nanoswitch calipers as a modular, broadly applicable approach for mechanostructural analysis of complex biomolecular systems.

## Graphical Abstract

**Graphical Abstract:**
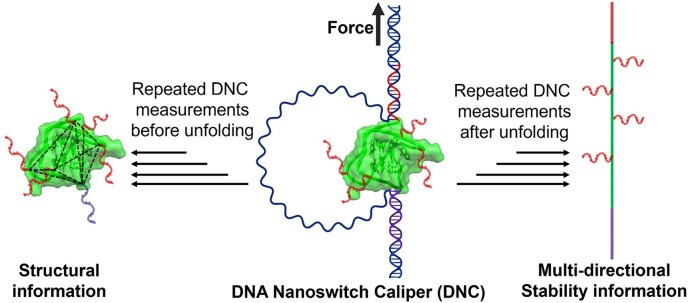

## Introduction

Under physiological conditions, biomolecules experience mechanical stresses that can alter their conformations, drive structural transitions such as unfolding or bond dissociation, and in turn modulate their functions [1, 2]. These responses often depend strongly on the direction of the applied force, highlighting the anisotropic nature of molecular mechanics [3–5]. Yet most experimental approaches isolate structure from mechanics, making it difficult to link force-induced conformational changes to function. Structural biology techniques such as Cryo-electron microscopy (cryo-EM), X-ray crystallography, and NMR spectroscopy provide high-resolution insights into molecular structures [6–8], with approaches such as pulsed EPR (DEER/PELDOR) [9] providing long-range distance constraints based on spin-labeling and ensemble averaging. Regarding single-molecule approaches, single-molecule fluorescence resonance energy transfer (smFRET) has been commonly used to determine the conformational dynamics of biomolecules [10], while single-molecule force spectroscopy methods such as AFM and optical tweezers probe their mechanical properties and stability [11–14]. These approaches offer valuable, complementary perspectives, though each has its limitations. Structural techniques often fail to capture mechanical properties, molecular heterogeneity and dynamics [15], while single-molecule studies generally provide limited structural insight, with distances and mechanical stability typically only measured along a single axis [10, 16], and labor-intensive engineering of multiple constructs generally needed to assess multidirectional mechanical stability.

**Figure 1.**
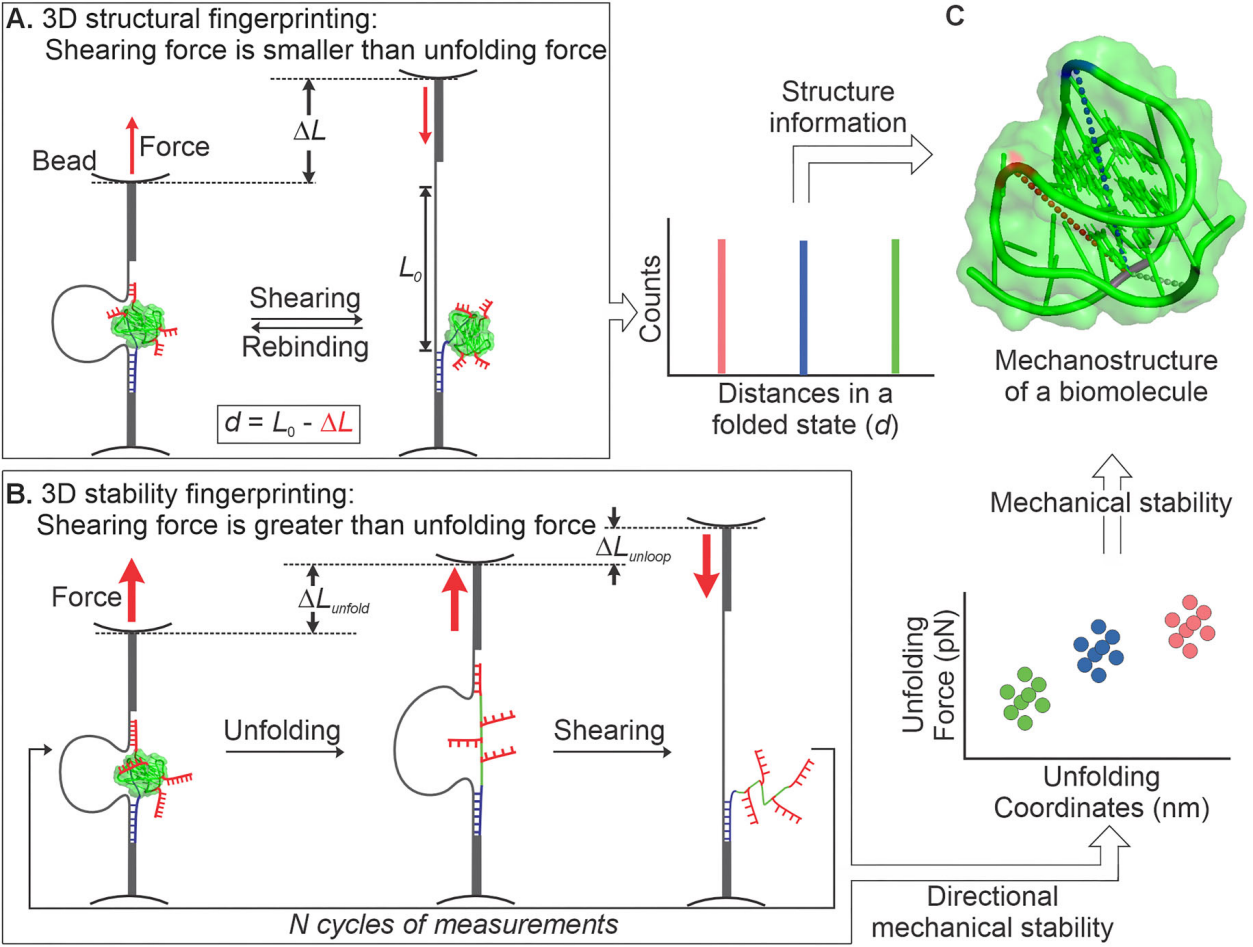
Schematic overview of mechanostructural fingerprinting using DNC. (**A**) Design of DNC for measuring multiple distances within a folded biomolecule. Shorter shearing handles are used to unloop the DNC before the structure unfolds. (**B**) Design of DNC for measuring the mechanical stability of a folded biomolecule along different pulling coordinates. Stronger shearing handles allow the structure to be unfolded prior to DNC unlooping, enabling directional unfolding along multiple axes. (**C**) Structural information from multiple distances in the folded state (A) combined with directional mechanical stability data (B) enables construction of a mechanostructural fingerprint of the folded biomolecule. Dotted lines depict the measured distances, and color coding reflects mechanical stability along different axes.

To address these gaps, an integrated approach is needed—one that combines structural and mechanical analyses of folded biomolecules. A technique capable of measuring multiple distances in a biomolecule to determine its 3D molecular geometry while enabling directional unfolding along various coordinates would provide a more comprehensive framework for investigating biomolecular mechanostructural properties and their roles in biological processes. Here, we present an advanced DNA nanoswitch caliper (DNC) approach [17, 18] that enables determination of both 3D molecular geometry and mechanical stability of folded biomolecules at the single-molecule level. DNC is a mechanically reconfigurable DNA device, developed in our labs by combining force spectroscopy with DNA structural nanotechnology, that uses single-molecule force spectroscopy to map multiple distances by mechanically grabbing and releasing single-stranded DNA handles labeled at specific residues of a target biomolecule (Fig. 1A). While earlier realizations of DNC measured structural distances, here we expand the platform to enable directional mechanical unfolding, integrating structural and mechanical characterization within a unified single-molecule framework. Thus, in contrast to established methods such as smFRET and EPR, our DNC approach can measure both absolute distances and changes in length at the single-molecule level under mechanical load, enabling multi-axis probing of structure and mechanics on the same molecule. Nevertheless, like other labeling-dependent techniques, DNC requires the introduction of specific attachment sites and explores only a finite set of pulling axes defined by these labeling positions.

As a proof of concept, we applied our approach to the human telomeric DNA G-quadruplex, a biologically significant yet labile structure formed by Hoogsteen hydrogen bonding between guanine bases and stabilized by specific metal cations. These structures can adopt distinct conformations, depending on nucleotide sequence, buffer conditions, and the identity of the stabilizing cations [19, 20]. Understanding the mechanical stability of these conformations is biologically and physiologically important, as G-quadruplexes can act as mechanical roadblocks that must be unfolded by motor proteins to allow continued replication or transcription. Similarly, RNA G-quadruplexes are known to regulate translation, and telomeric G-quadruplexes can regulate telomerase activity and influence telomere length [14, 21–23]. We demonstrate that DNC enables the determination of both G-quadruplex conformation, through measurement of multiple intramolecular distances in the folded state (Fig. 1A), and anisotropic mechanical stability, through directional unfolding along multiple axes (Fig. 1B), producing a distinct mechanostructural fingerprint (Fig. 1C). This study establishes DNC as a simple yet modular nanoscale platform for mechanostructural analysis of biomolecules and their complexes, complementing high-resolution structural biology techniques with insights into molecular stability and mechanical anisotropy, and paving the way toward understanding single-molecule conformational heterogeneity. Furthermore, as detailed below, the reproducibility and reliability of the method can be characterized and improved through calibration using known reference constructs and by the use of robust data analysis procedures, including per-molecule averaging, bootstrap-based uncertainty quantification, and complementary likelihood analysis.

## Materials and methods

### Materials

The pET-26b (+) plasmid was received from EMD Millipore Sigma. All the DNA oligonucleotides (see sequences in Supplementary Table S1) were purchased from Integrated DNA Technologies. The enzymes required to synthesize DNC constructs were purchased from New England Biolabs. Chemicals such as 1-Ethyl-3-(3-dimethylaminopropyl) carbodiimide and N-hydroxysuccinimide ester were purchased from Sigma Aldrich. The carboxylated silica beads (3 µm) were purchased from Microspheres-Nanospheres.

### Site-specific labeling of G-quadruplex with single-stranded DNA handles

The single-stranded DNA oligo with a human telomeric sequence (TTAGGG)_4_ that had an azide functional group in the first thymine base of each loop sequence (TTA) was incubated with the DNA handle labeled with DBCO at the 5′ end. The reaction mixture was prepared in 1× Phosphate Buffered Saline (PBS) buffer and sealed with paraffin film and incubated for >18 h in no direct light. The completion of the reaction was tested in polyacrylamide gel electrophoresis assay and the fully labeled target was purified from the gel (Supplementary Fig. S1).

### Synthesis of DNA nanoswitch caliper constructs

Two different DNC constructs were synthesized, one incorporating a short loop and the other a long loop, following previously described methods [17, 18]. Briefly, the long double-stranded DNA (dsDNA) handle (2820 bp) used in both constructs was prepared by polymerase chain reaction (PCR) amplification of a specific region of the pET-26b (+) plasmid. The forward primer contained a BsaI restriction site, and the reverse primer was labeled with dual biotins at the 5′ end (see sequences in Supplementary Table S1). The PCR product was purified using a PCR purification kit (Qiagen) and then digested with BsaI restriction enzyme to create sticky ends for ligation with the other fragment of the DNC construct. The other DNC fragment was prepared by annealing the loop oligonucleotide (short or long loop), 5′-digoxigenin modified oligonucleotide, and other required strands (Supplementary Table S1) at equimolar concentration with a splint oligonucleotide added at 10-fold molar excess. The final DNC construct was synthesized by ligating this annealed fragment to the dsDNA handle using T4 DNA ligase (New England Biolabs) at 16°C for 16 h, followed by heat deactivation of the ligase at 65°C for 20 min. The fully ligated product was purified using agarose gel electrophoresis.

### Single-molecule distance and unfolding force measurements using optical tweezers

We used home-built dual-trap optical tweezers for all DNC measurements [24]. The short-loop DNC was used to measure distances in the folded G-quadruplex, while the long-loop DNC was used to unfold the structure along multiple different pulling axes. All DNC measurements were performed in Tris-buffered saline (150 mM NaCl or 150 mM KCl, 10 mM Tris buffer, pH 7.4) with 0.1% (w/v) Roche Blocking solution (Roche) at room temperature (23°C). First, 5 µl of DNC construct (~100 pM) was incubated with 0.5 µl of a 1% (w/v) solution of streptavidin-coated silica beads (3.0-µm diameter) for 30 min to immobilize the DNC construct on the surface of the beads through binding of biotin and streptavidin. The DNC-functionalized beads were washed twice by centrifugation to remove unbound constructs. For an end-to-end distance measurement in a folded G-quadruplex, the target DNA consisting of a human telomere G-quadruplex sequence sandwiched between a grabbing handle sequence at the 5′ end and a shearing handle sequence at the 3′ end was incubated with the DNC immobilized beads for 30 min. Then, the beads were washed to remove excess target DNA oligonucleotides. To measure multiple distances in an individual G-quadruplex, G-quadruplex labeled with shearing handles in each loop site (as prepared above), was incubated with the DNC immobilized beads for 30 min, followed by washing to remove the unbound targets.

**Figure 2.**
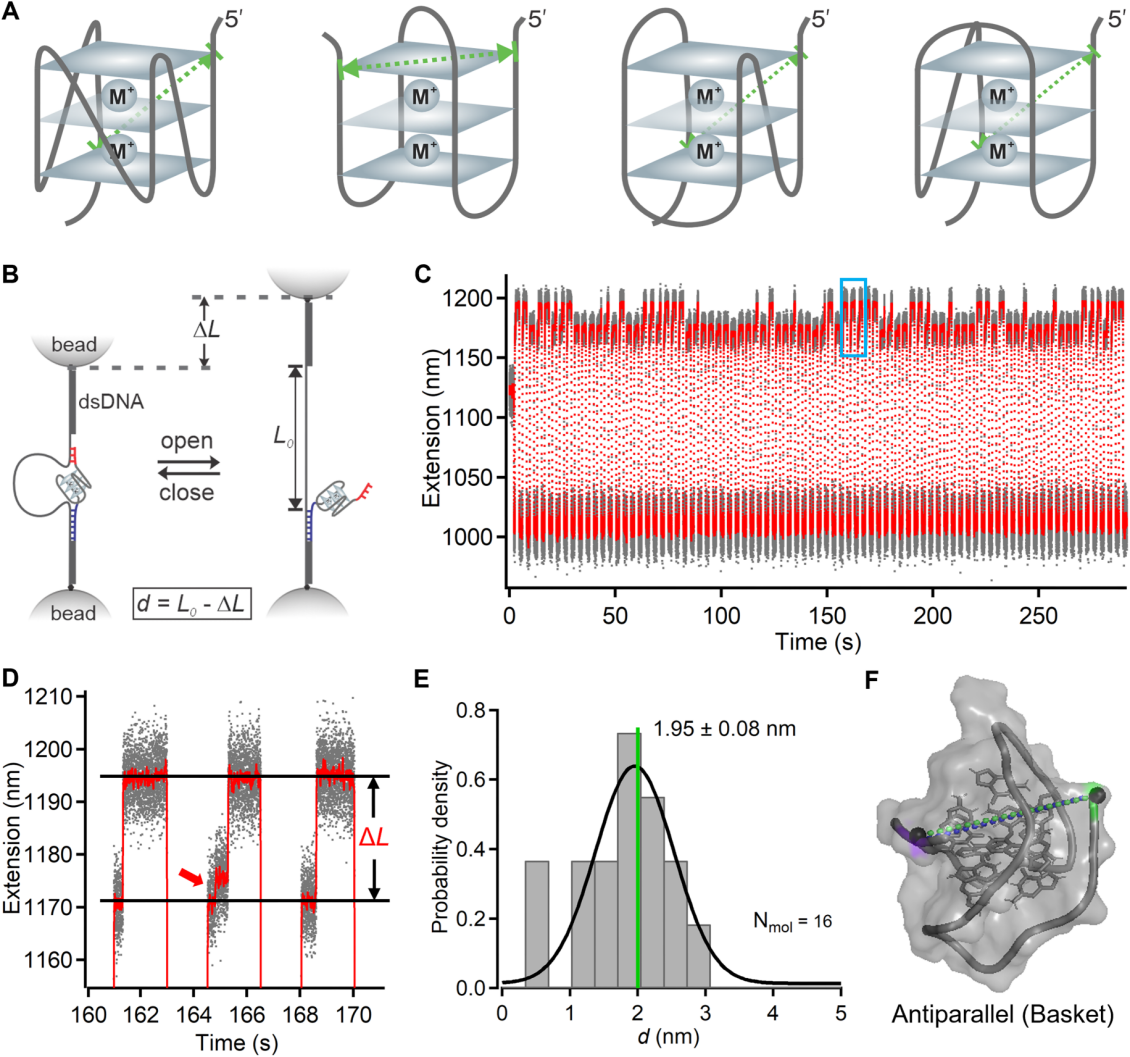
Single-molecule measurements of 5′-3′ distance in a folded G-quadruplex in sodium buffer. (**A**) Schematic structures of four representative G-quadruplex conformations. Left to right: Parallel, Antiparallel, Hybrid 1, and Hybrid 2. Green arrows indicate end-to-end distances. (**B**) Schematic depiction of the measurement strategy using DNC and optical tweezers. The 5′ handle (purple) serves as a strong grabbing handle, and the 3′ handle (red) serves as a weak shearing handle. Unlooping of DNC is recognized as a change in length (Δ*L*). The absolute distance (*d*) is calculated by subtracting Δ*L* from the loop length of the DNC (*L_0_*). (**C**) Representative extension versus time graph showing Δ*L* measurements obtained by jumping the force between ~10 and 0 pN in repeated cycles. Gray trace shows raw data, while the red trace indicates sliding-window averaged data (window size = 200). (**D**) Zoomed-in view of the blue rectangle section in panel (C), showing repeated distance measurements and occasional unfolding events (red arrow) during force cycles. (**E**) Histogram of per-molecule averaged 5′-3′ (end-to-end) distances for 16 G-quadruplexes. Plus-minus values indicate the standard error of the mean. The blue vertical line indicates the NMR-determined distance for the antiparallel (basket) conformation. N_mol_ denotes the number of measured molecules. (**F**) Comparison of the 5′-3′ distance measured by DNC (green dotted line) and that reported by NMR (PDB 143D, blue dotted line) for the antiparallel (basket) conformation showing strong agreement.

**Figure 3.**
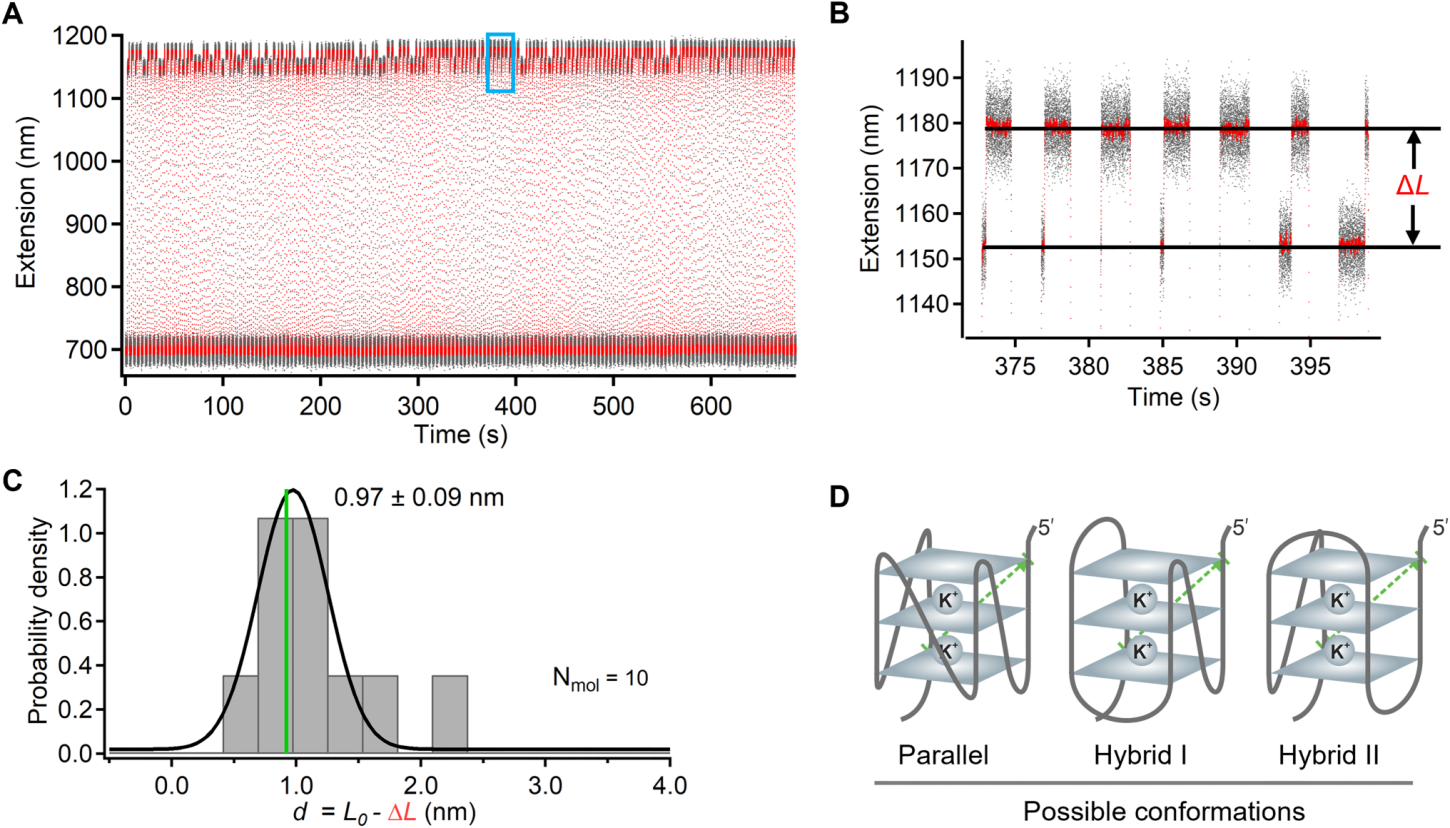
Single-molecule measurement of 5′-3′ distance in a folded G-quadruplex in potassium buffer. (**A**) Representative extension versus time graph showing Δ*L* measurements obtained by cycling the force between ~10 pN to shear the red handle and ~0 pN to enable handle rebinding. Gray trace shows raw data, while the red trace indicates sliding-window averaged data (window size = 200). (**B**) Zoomed-in view of the blue rectangular region in panel (A), showing repeated distance measurements in the folded G-quadruplex. (**C**) Histogram of per-molecule averaged distances for 10 G-quadruplexes. Plus-minus values indicate the standard error of the mean from Gaussian fitting. The green vertical line indicates the 5′-3′ distance from the NMR-determined structure. (**D**) The measured 5′-3′ distance is consistent with three possible conformations reported in structural studies.

**Figure 4.**
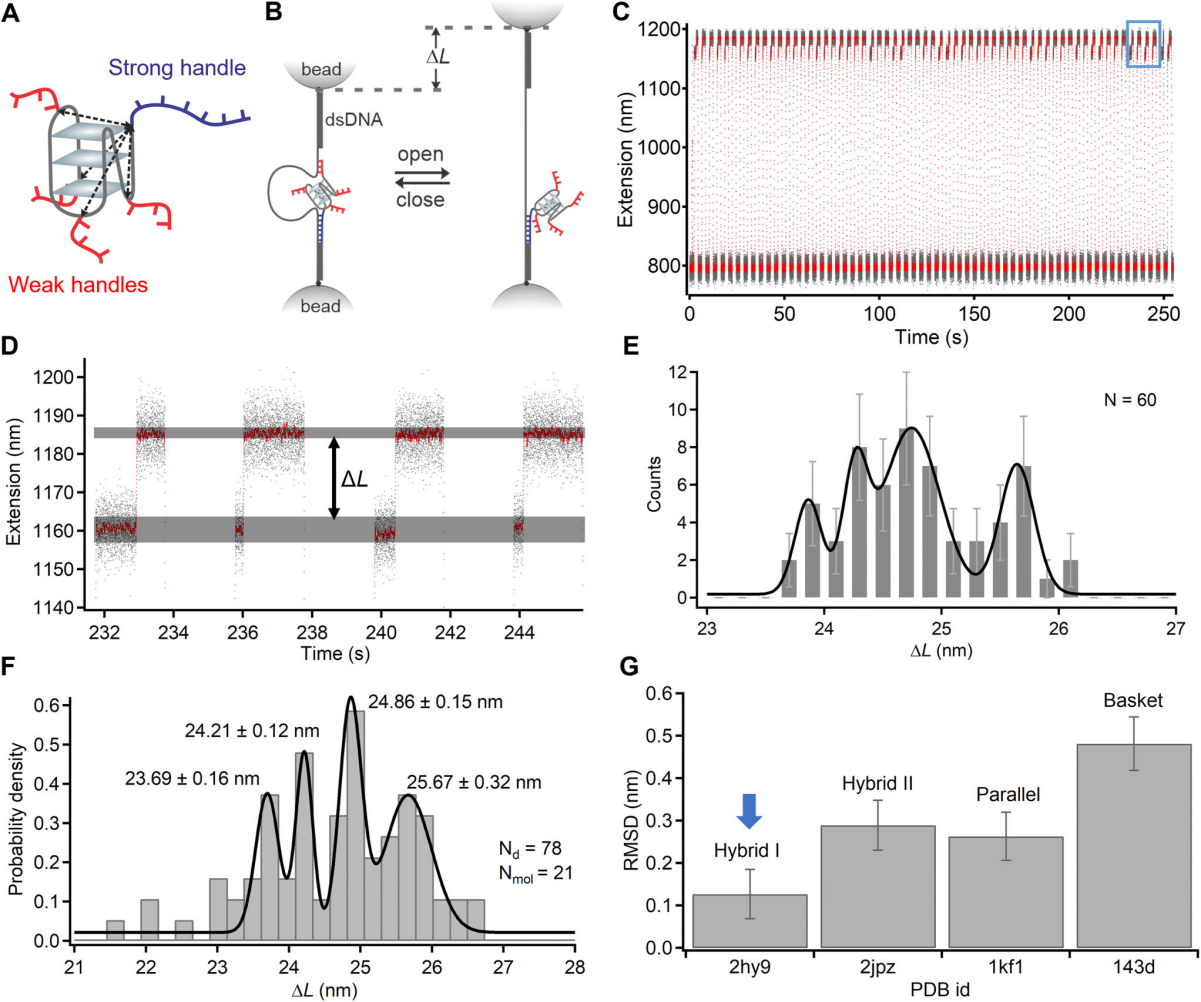
Measurement of multiple distances in a folded G-quadruplex in potassium buffer. (**A**) Schematic representation of the G-quadruplex structure showing the four pairwise distance measurements (dashed arrows). One strong grabbing handle (purple) is positioned at the 5′ end, and four weak shearing handles (red) are attached at three internal loop positions and at the 3′ end. (**B**) Schematic of the DNC measurement strategy using optical tweezers. (**C**) Raw extension versus time data showing the change in extension over multiple measurement cycles (gray), red trace indicates sliding-window averaged data (window size = 200). (**D**) Zoomed-in view of the blue rectangular region in panel (C) showing unlooping events corresponding to different distances within the folded G-quadruplex. Horizontal gray bands indicate the states before and after DNC unlooping. (**E**) Histogram of change in length (Δ*L*) values measured in a single G-quadruplex. (**F**) Histogram of per-molecule averaged Δ*L* values measured in 21 G-quadruplexes. Plus-minus values indicate sigma fitting parameters from the multiple Gaussian fit. (**G**) Root-mean-squared deviation (RMSD) analysis comparing measured Δ*L* values to those expected for four representative G-quadruplex conformations.

**Figure 5.**
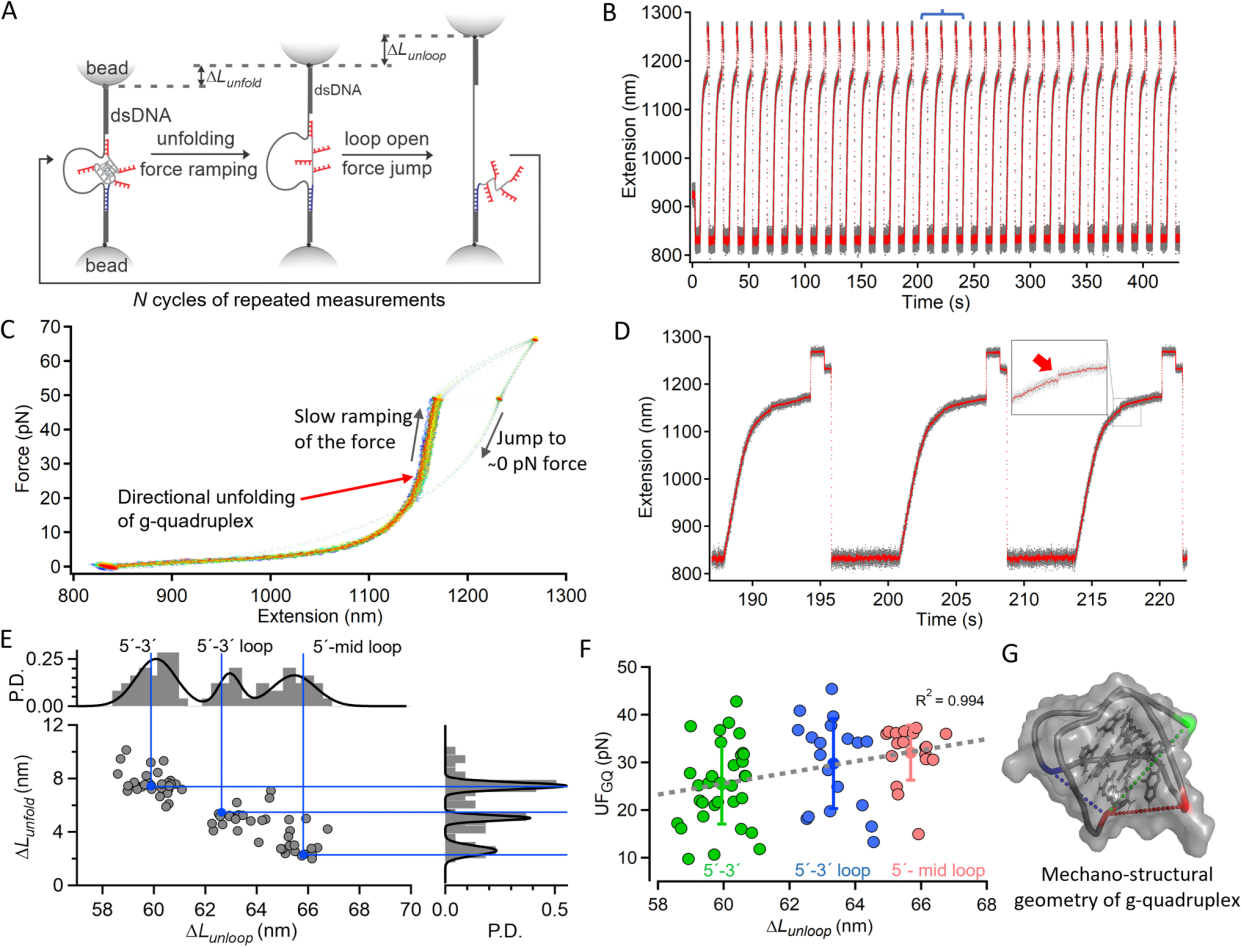
Multidirectional unfolding of individual G-quadruplex structures. (**A**) Schematic of design and experimental strategy to unfold single G-quadruplexes along multiple pulling directions. First step, slow force ramp to probe unfolding, step 2, force jump to shear-open the DNC and identify the unfolding site. (**B**) Experimental extension versus time trace (gray = raw, red = sliding-window average, window = 100). (**C**) Overlapped force-extension curves from repeated measurements of the same G-quadruplex, spread region reflects multiple unfolding cycles. (**D**) Zoomed section (blue in panel B) showing unfolding features (red arrow at the inset). (**E**) Scatter plot of Δ*L*_unfold_ from G-quadruplex unfolding and DNC unlooping, Δ*L*_unloop._ Top and right: histograms of Δ*L*_unfold_ and Δ*L*_unloop_ with multi-peak Gaussian fits (black), blue lines mark expected Δ*L* values. (**F**) Correlation between unfolding force and pulling direction. Color codes indicate force clusters from different directions (defined by Δ*L*_unloop_). Gray dotted lines show linear fits to mean values. (**G**) Mechanical stability of G-quadruplex mapped onto the NMR structure in Na^+^ (PDB 143D), consistent with DNC measurements. Color-coded residues indicate pulling directions in panel (F), dashed lines show inter-residue distances.

The sample cell was prepared as described previously [17, 18]. Briefly, a double-sided Kapton tape (1 mm, DuPont) was sandwiched between a glass slide and a cover glass (prewashed with 70% ethanol and dried with argon flow). Two side channels were created by cutting the Kapton tape before sandwiching for injecting DNC immobilized beads and anti-digoxigenin-coated beads. The channel was passivated with Roche Blocking solution, [1% (w/v)] for ~30 min and then flushed with the experimental buffer before injecting the beads. The DNC immobilized beads (1 µl) were injected through one side-channel and anti-digoxigenin coated silica beads [1 µl of the 0.1% (w/v)] were injected through the next side-channel. The channel openings were then sealed with grease to prevent dry-up of the sample during measurements. In dual-trap optical tweezers, the two different beads were trapped in separate laser foci and a tether was formed by binding digoxigenin at the end of the immobilized DNC with an antidigoxigenin-coated bead as shown in Figs 2–5. The tethered DNC was confirmed as a single molecule by estimating the contour length of the whole caliper (~1 µm) and by a single breakage of the tether or the observation of a ~65 pN plateau in force due to mechanical overstretching of dsDNA. The distance measurement on the folded G-quadruplex was carried out by shearing the handle at a low force (~10 pN, which is less than the approximate unfolding force of the G-quadruplex of ~21 pN) and then jumping to a force of ~0 pN to let the shearing handle rebind. By repeatedly cycling between force levels, extensive data was collected from each individual caliper. For directional unfolding of the G-quadruplex, a G-quadruplex labeled with the longer shearing handles (see Supplementary Table S1) was used. Slow ramping of the force (~6 pN/s) up to ~45 pN was used to probe unfolding of the G-quadruplex, followed by a rapid jump to a higher force to unloop the DNC to enable identification of the attachment site on the construct. Data was recorded at 1400 Hz using custom LabVIEW 2013 software (National Instruments Corporation, Austin, TX). Briefly, LabVIEW programs were written to carry out both dynamic force spectroscopy, in which force is gradually increased, and constant force measurements. For conformation identification experiments, only the force-jump procedure was used, while multidirectional unfolding experiments first used dynamic force spectroscopy to probe unfolding, followed by a force jump to unloop the DNC.

### Data analysis

For all single-molecule measurements, data was acquired using custom LabVIEW software and analyzed with custom MATLAB scripts. Statistical analyses and graphing were performed using IGOR Pro. G-quadruplex unfolding was identified by a sudden change in extension during slow ramping of the force (Fig. 5), whereas DNC unlooping was determined by a discrete change in length (Δ*L*) during the holding period at the shearing force (Figs 2–5). Since unfolding of the structure was measured using a dynamic force spectroscopy approach, the change in length associated with unfolding (Δ*L*_unfold_) was obtained by fitting the force-extension curves before and after unfolding using the worm-like chain model [25] and calculating the difference in contour length. In contrast, the change in length associated with DNC unlooping (Δ*L*_unloop_) was determined from the increase in extension resulting from unlooping at constant force. To convert the observed Δ*L* from DNC unlooping into absolute distance *d*, we subtracted Δ*L* from the effective loop length *L_0_*, i.e. *d* = *L_0_* − Δ*L*. It is important to account for the length compliance from the additional nucleotide linkers when calculating *d. L_0_* at the measurement force was determined by measuring Δ*L* for analytes of known length and performing a linear regression to extrapolate the change in length for a zero-length analyte, i.e. the offset of the linear fit is *L_0_*. However, for multi-distance and directional unfolding measurements, we interpreted results directly in terms of Δ*L, as L_0_* varied across configurations due to differences in the linkers in the loop-region shearing handles compared to those at the 3′- end.

To precisely estimate the measured values of multiple distances, Gaussian multi-peak fitting was applied first to the change in length (Δ*L)* data from individual molecules, followed by characterization of the per-molecule average distribution, and then bootstrapping analysis [26, 27]. For each molecule, the Δ*L* measurements were binned. Next, distinct peaks were identified in the Δ*L* histograms and used as initial parameter guesses for multi-peak Gaussian fitting performed in Igor Pro. The means of the resulting peak positions from individual molecules were then combined to form a per-molecule average distribution, which was analyzed using Gaussian multi-peak fitting to determine the peak positions within this population of molecules. To access uncertainty, we performed a bootstrapping analysis using 10 000 iterations of parametrically resampled values using the means and standard deviations of the fit distributions, followed by Gaussian multi-peak fitting of each resampled data set. Finally, fitted peak positions were compared to distances expected from G-quadruplex structures published in the PDB to calculate RMSD, with associated errors quantified using standard error propagation methods.

To further validate the identification of the most likely G-quadruplex structure, we applied maximum likelihood modeling using Gaussian mixture models, following the approach of Shrestha *et al.* [17]. For each of the four possible G-quadruplex structures, we generated a probability distribution consisting of Gaussians centered at the expected mean positions of the four measurement sites. The Gaussian standard deviation was fixed at 0.5 nm, based on the observed variance of DNC measurements obtained by optical tweezers, and we assumed a uniform probability of sampling from the four measurement sites. For each set of caliper measurements, we calculated the likelihood that the observed data arose from each candidate structure’s probability distribution. These likelihoods were normalized across the four possible structures for each single-molecule measurement set and then averaged over all 21 molecules to yield the final probability for each structure (Supplementary Fig. S7).

### Circular dichroism spectroscopy

Human telomere DNA oligonucleotide (Supplementary Table S1) samples were prepared at 5 µM in 10 mM Tris buffer (pH 7.4) containing either 150 mM NaCl or 150 mM KCl. Samples were first heated at 95°C for 5 min, then rapidly cooled in an ice bath, followed by incubation at room temperature for up to an hour. CD spectroscopy was performed in a 1 mm quartz cuvette at room temperature with a JASCO J-1500 spectropolarimeter. Scans were performed from 200 nm to 320 nm at a rate of 100 nm/min. Reported spectra represent the average of the recorded data using a window size of 100. The spectrum of the corresponding buffer was used for baseline correction.

## Results and discussion

### Measurement of the distance between 5′ and 3′ ends in a folded G-quadruplex structure in different ionic conditions

DNA G-quadruplexes are polymorphic, with conformations governed by the type of stabilizing cations and the nucleotide sequences [28–31]. There are different types of DNA G-quadruplex conformations such as parallel, anti-parallel, and mixed-type hybrid-1 and hybrid-2. Depending on the folded G-quadruplex conformation, the 5′ to 3′ end-to-end distances can vary (Fig. 2A).

We confirmed that the target human telomere sequence adopts distinct conformations in sodium and potassium buffers using circular dichroism spectroscopy [32] (Supplementary Fig. S2). To measure the end-to-end distance in the folded state at the single-molecule level, we designed a short (9 base) DNA handle at the 3′ end that shears open the DNC at ~10 pN, below the ~20 pN unfolding force of the G-quadruplex [11, 17]. First, we measured the end-to-end distance of the folded G-quadruplex in sodium buffer (10 mM Tris, pH 7.4, 150 mM NaCl) as shown in Fig. 2B. In repeated force cycles, we jumped the force between two different levels—~10 pN to shear open the DNC, indicated by a sudden change in extension (Δ*L*) (Fig. 2 B and C), and ~0 pN to allow the handle to rebind, enabling repeated distance measurements within the same molecule. At the shearing force, G-quadruplex unfolding sometimes occurred prior to DNC unlooping, as indicated by the red arrow in Fig. 2D, and hence the histogram of the change in length measured from several molecules showed three major populations: ~6.0 nm due to unfolding of the G-quadruplex, ~20.0 nm due to unlooping from the unfolded G-quadruplex, and ~26.0 nm due to unlooping from the folded G-quadruplex (Supplementary Fig. S3). By subtracting the change in length Δ*L* associated with DNC unlooping in the folded G-quadruplex state from the loop length [18], we obtained the distribution of the measured end-to-end distance of the folded G-quadruplex, which indicated a single population centered at ~2.0 nm (Fig. 2E). This distance of ~2.0 nm measured using DNC matches that of the antiparallel conformation reported by NMR [33, 34], confirming that the folded G-quadruplex adopts this conformation in these sodium buffer conditions (Fig. 2F). Single-molecule FRET measurements have also reported the antiparallel conformation as a major population in sodium buffers but also observed a certain extent of conformational heterogeneity suggesting that multiple interconvertible states could exist under different conditions [35].

On the other hand, when we switched from sodium to potassium buffer (10 mM Tris, pH 7.4, 150 mM KCl), the measured 5′-3′ (end-to-end) distance distribution shifted to ~1.0 nm, indicating that the G-quadruplex adopted one of several alternative conformations (Fig. 3 and Supplementary Fig. S4). Three candidate structures, each with similar end-to-end distances, have been reported in NMR studies [36, 37] (Supplementary Table S2). Distinguishing between these conformations requires the measurement of multiple distances within the folded G-quadruplex. Two distinct conformations in sodium and potassium ion buffers are also supported by the differential occurrence of unfolding events at the unlooping force (Figs 2D and 3B). In sodium ion buffer, we occasionally observed G-quadruplex unfolding while waiting for DNC unlooping, whereas no such unfolding was detected in potassium ion buffer. This difference aligns with previous findings that the G-quadruplex conformation in sodium exhibits lower mechanical stability than the corresponding conformation in potassium [38, 39].

### Measurement of multiple distances in a folded G-quadruplex in potassium buffer

Next, to resolve between the three potential conformations of the G-quadruplex in potassium buffer, we utilized the capability of DNC to measure multiple distances within the same biomolecule [17, 18]. A G-quadruplex with multiple shearing handles was prepared by labeling the first thymine base in each loop region using DBCO-azide click chemistry (Supplementary Fig. S1). We measured pairwise distances between the 5′ end and each of the three loop-labeled handles, as well as the 3′ end of each folded structure. As before, we performed these measurements by cycling between a shearing force of ~10 pN and a rebinding force of ~0 pN to enable sampling of all four distances (Fig. 4 A–E). The distribution of the change in length (Δ*L)* values showed multiple peaks, as expected (Fig. 4E and Supplementary Fig. S5). After per-molecule averaging, four prominent peaks were evident in the resulting histogram, consistent with successful measurement of all four distances in the folded G- quadruplex (Fig. 4F and Supplementary Fig. S6).

To identify the most likely conformation, we compared the measured distances to those expected from published NMR structures and calculated the RMSD for each (Supplementary Table S3). Based on the lowest RMSD, the conformation of the G-quadruplex in potassium buffer is most consistent with the hybrid I (PDB 2HY9) conformation (Fig. 4G). Additionally, we performed a complementary maximum likelihood analysis of the DNC-measured distances to determine the most probable of the expected four G-quadruplex conformations (Fig. 2A). As described in the methods, the analysis was based directly on the measured distances, without relying on histogram binning or curve fitting. This analysis was performed on a per-molecule basis, then averaged to determine the most probable G-quadruplex conformation under these buffer conditions. This alternative probabilistic analysis also supported the hybrid I conformation (Supplementary Fig. S7), consistent with our original assignment, which also matches previous reports [40]. These results highlight the capability of DNC to mechanically probe and distinguish between conformations of folded biomolecular structures.

G-quadruplex structures are composed of rigid guanine-quartet (G-quartet) cores and more flexible loop regions, with loop length known to influence conformation and dynamics [41]. In our DNC assay, distance measurements involving loop regions may be influenced by their structural compliance. Notably, in human telomere G-quadruplex, each loop contains only three nucleotides. To minimize potential measurement variability arising from flexible loops, we strategically applied force through the nucleotide closest to the G-quartet, likely reducing measurement noise. Nevertheless, we observed heterogeneity in distance histograms between individual molecules.

Single-molecule methods uniquely facilitate the characterization of population heterogeneity at the single-molecule level [42]. The variability we observed in distance histograms (e.g. Supplementary Fig. S5), likely reflects factors such as labeling variability, limited sampling in some molecules, or mixtures of multiple distinct conformations (i.e. static heterogeneity). Although our force conditions were specifically chosen to minimize molecular unfolding and subsequent conformational switching between measurements, minor contributions from dynamic heterogeneity (conformational transitions within a single molecule) cannot be entirely excluded. Future studies could further dissect these contributions through expanded single-molecule data sets that incorporate complementary information, such as simultaneous single-molecule FRET measurements, and through advanced force-spectroscopy analyses such as nonparametric Bayesian inference [42].

### Directional unfolding of individual G-quadruplex structures in sodium buffer

Biomolecular folding is governed by a complex network of intramolecular interactions—such as hydrogen bonding, base stacking, and electrostatic contacts—that are oriented in specific spatial arrangements within the folded structure. Because these interactions are anisotropic, the mechanical stability of a folded biomolecule is dependent on the direction from which force is applied. Mechanical unfolding using single-molecule techniques enables directional probing of molecular stability, distinct from the more global unfolding induced by chemical denaturants or changes in temperature. This approach has provided valuable insights into kinetic intermediates and folding pathways in biomolecules [43–46]. However, such measurements are often challenging, as single-molecule mechanical unfolding is typically performed along a single pulling direction per construct, requiring multiple different designs to interrogate direction-dependent behavior [16, 27, 44, 47]. Here, we demonstrate how DNC can be used to unfold individual G-quadruplex structures in multiple different directions. To enable this, we designed G-quadruplex constructs with longer shearing handles (16 bases) at the 3′ end (Supplementary Fig. S8 and Supplementary Table S1), as well as in each loop region, labeling the first thymine base in each loop region using DBCO-azide chemistry. These longer handles were designed so that unlooping of the DNC would occur at a higher force than G-quadruplex unfolding, allowing us to observe first unfolding, then opening of the DNC to identify which handle was grabbed and know the direction of unfolding. During each experimental cycle, unfolding of the G-quadruplex was observed as a small, sudden change in extension (Δ*L*_unfold_) of ~2–9 nm during a period of slow force ramping up to ~45 pN. The force was then rapidly increased to > 55 pN to shear open the DNC, producing a larger change in extension (Δ*L*) of ~58–68 nm, which revealed the handle through which the G-quadruplex had been unfolded (Fig. 5A–D). We observed unfolding along three distinct pulling directions (5′-end to mid-loop, 5′-end to the 3′-end loop, and 5′-end to the 3′-end) as confirmed by the expected Δ*L*_unfold_ and Δ*L* values within each cycle (Fig. 5E and Supplementary Table S4). As expected, we did not observe unfolding through the 5′-end loop, as the expected change in distance due to unfolding was below our detection threshold in this pulling direction (Supplementary Table S4). When comparing unfolding forces across different pulling directions, we found that pulling through the loops required a slightly higher force than pulling from the 5′-end to the 3′-end (Fig. 5F). This may reflect differences in the alignment of Hoogsteen H-bonding relative to the pulling direction: unfolding from 5′ to 3′ proceeds along the stacking axis in an unzipping-like mode, whereas pulling through the loops engages the G-quadruplex at more oblique angles, which may require higher forces for disruption.

By combining the measurement of distances in the folded state (Fig. 2) with directional unfolding data (Fig. 5), we generated a mechanostructural fingerprint of the G-quadruplex conformation (Fig. 5G), providing an integrated picture of its structure and mechanical anisotropy. Recent work by Yang *et al. *[5] and Sedlak *et al. *[4] demonstrate that mechanical stability in receptor-ligand complexes can strongly depend on tethering geometry, establishing that the direction of force application alone can modulate unbinding behavior. Our results extend this concept to folded nucleic acid structures, revealing that the direction of applied force can significantly influence unfolding behavior. Such directional sensitivity could impact how effectively G-quadruplex structures act as mechanical roadblocks during replication and transcription, and how telomerase engages telomeric DNA substrates. By enabling directional unfolding and multipoint distance measurements within the same molecule, DNC overcome the key limitations of previous single-molecule approaches. This method will provide a powerful tool for probing mechanostructural relationships in nucleic acids and their protein complexes, enabling direct investigations into how structural conformations respond to biologically relevant mechanical forces.

## Supplementary Material

gkaf1465_Supplemental_File

## Data Availability

The data underlying this article are available in the manuscript and its online supplementary material. Raw data of optical tweezers and circular dichroism spectroscopy measurements are available on Zenodo using digital object identifiers as https://zenodo.org/records/15664467. The codes used to analyze the data are available at https://github.com/pshresthalab/Shrestha-et-al-Nucleic-Acids-Research-2025.
