# Supplementary material for: Single-molecule mechanostructural fingerprinting of nucleic acid conformations
Source: Nucleic Acids Res. 2026 Jan 6;54(1):gkaf1465. doi: 10.1093/nar/gkaf1465 (PMC12774654; doi:10.1093/nar/gkaf1465)
Supplement: gkaf1465_Supplemental_File [file gkaf1465_supplemental_file.pdf]

Supplementary Information for

Single-molecule Mechanostructural Fingerprinting of Nucleic Acid Conformations

Prakash Shrestha[1,2,3,5*], Hans T. Bergal[2,5], William M. Shih[3,4,5], and Wesley P. Wong[2,3,5,6*]

[1] Department of Chemistry, University of Kentucky

[2] Program in Cellular and Molecular Medicine, Boston Children's Hospital.

[3] Wyss Institute for Biologically Inspired Engineering, Harvard University.

[4] Department of Cancer Biology, Dana-Farber Cancer Institute.

[5] Department of Biological Chemistry and Molecular Pharmacology, Blavatnik Institute, Harvard Medical School.

[6] Department of Pediatrics, Harvard Medical School.

*Correspondence: prakash_shrestha@uky.edu, Wesley.Wong@childrens.harvard.edu

Supplementary Table 1. List of DNA oligonucleotides that are used to synthesize DNA Nanoswitch Calipers (DNC), target DNA oligonucleotides and DNA handles used to label the G-quadruplex. Purified DNA oligonucleotides were purchased from IDT.

**Oligonucleotides**	**Sequences**
BsaI-forward primer	AAA AAA GGT CTC GGC ATG ATA GCG CCC GGA AGA GAG
Biotin-reverse primer	/52-Bio/GGG TTC GTG CAC ACA GCC CAG CTT
Loop_short	ATG CCG CGT AAC CGG TCC GAG ACT CTC ACT GTG AAC TGA GTT TTT TTT TTT TTT TTT TTT TTT TTT TTT TTT TTT TTT TTT TTT TTT TTT TTT TTT TTT TTT TTT TTT TTT TTT TTT TCA GCG TCC GCG TGT CAG TGT
Loop_long	ATG CCG CGT AAC CGG TCC GAG ACT CTC ACT GTG AAC TGA GTT TTT TTT TTT TTT TTT TTT TTT TTT TTT TTT TTT TTT TTT TTT TTT TTT TTT TTT TTT TTT TTT TTT TTT TTT TTT TTT TTT TTT TTT TTT TTT TTT TTT TTT TTT TTT TTT TTT TTT TTT TTT TTT TTT TTT TTT TTT TTT TTT TTT CAG CGT CCG CGT GTC AGT GT
DNC1	/5DigN/CGT CCC CAA ATC CGT ACC GGA TGC ACT GGC ACT GTG GGT GTG CGA CTT AAT TCC ATC CTG TTC CTG AGA CAA TAC AGC ACG ACC GCT TTC
DNC2	/5Phos/TCT AAC TTA CAG AGC ATG GCG AAA GCG GTC GTG CTG TAT TGT CTC AGG AAC AGG ATG GAA TTA AGT CGC ACA CCC ACA GTG CCA GTG CAT CCG GTA CGG ATT TGG GGA CG
DNC3	TCT CGG ACC GGT TAC GCG
Splint	AAG TTA GAA CAC TGA C
DNC4	GAT GGC CCG CTG AAA GGG CAG TGT TTC CCA GCG CCC TTC CTG GTA TGC GGA TTC TTT CGG GAG ATA GTA ATT

	AGC ACC TCC ATC GTC AGA CGG CGT CTG TGG CTC CGG CCT GAA CAG TGA
DNC5	/5Phos//TCT AAC TTA CAG AGC ATG GCT CAC TGT TCA GGC CGG AGC CAC AGA CGC CGT CTG ACG ATG GAG GTG CTA ATT ACT ATC TCC CGA AAG AAT CCG CAT ACC AGG AAG GGC GCT GGG AAA CAC TGC CCT TTC AGC GGG CCA TCG TGA TTT GAC TGT CTC CGG CCT TTC TCA CCC TTT TGA ATC TTT ACC TAC ACA TTA CTC AG
GH-GQ-LM	GCC ATG CTC TGT AAG TTA GAA CAC TGA CAC GCG GAC GCT GTT AGG G/iAzideN/T AGG G/iAzideN/T AGG G/iAzideN/T AGG GTT ACT CAG TTC A
GH-GQ-9bSH	GCC ATG CTC TGT AAG TTA GAA CAC TGA CAC GCG GAC GCT GTT AGG GTT AGG GTT AGG GTT AGG GTT ACT CAG TTC A
GQ-9bSH	/5DBCON/CTC AGT TCA
GH-GQ-9bSH-ext	/5Phos/CAG TGA G
Ext-splint	CAT GTC GAC TCA CTG TGA ACT GAG T
Ext-splint-removal	ACT CAG TTC ACA GTG AGT CGA CAT G
GQ-16bSH	/5DBCON/CTC AGT TCA CAG TGA G

Sequences from left to right are in the 5′ to 3′ directions. Underlined sequence represents the human telomere sequence (TTAGGG)$_4$ that can fold into a G-quadruplex structure.

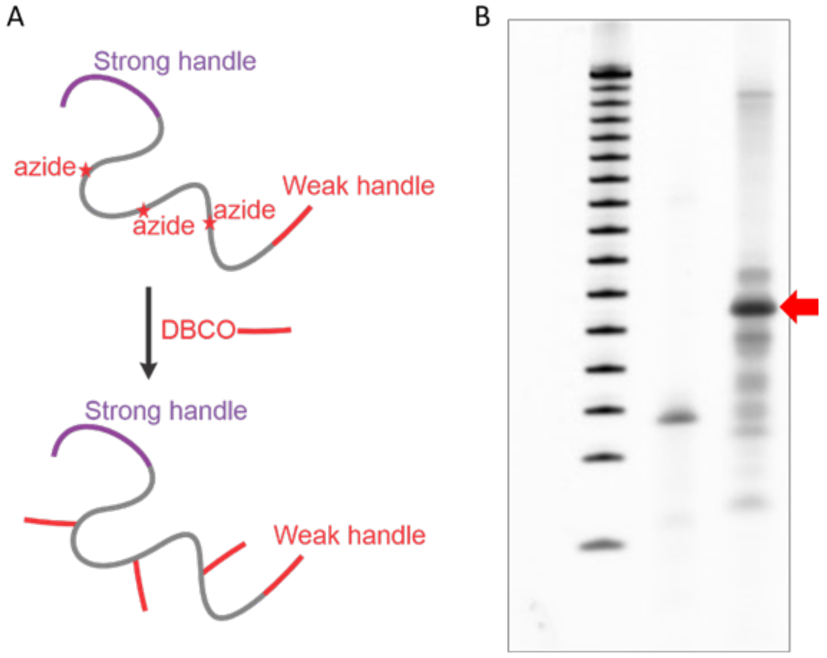

Supplementary Figure 1. Labeling of a nucleotide in each loop of G-quadruplex loop with a shearing handle. A) Schematic representation of the DBCO-azide click reaction to label shearing handles in each loop of the g-quadruplex. B) PAGE analysis to confirm the successful labeling reaction. Left Lane: 50 bp ladder (Promega), Middle Lane: ssDNA consisting of a G-quadruplex forming sequence, and Right Lane: ssDNA consisting of a G-quadruplex forming sequence with a shearing handle attached in each loop region of G-quadruplex. The red arrow indicates the expected product that was gel purified to use in DNC measurements.

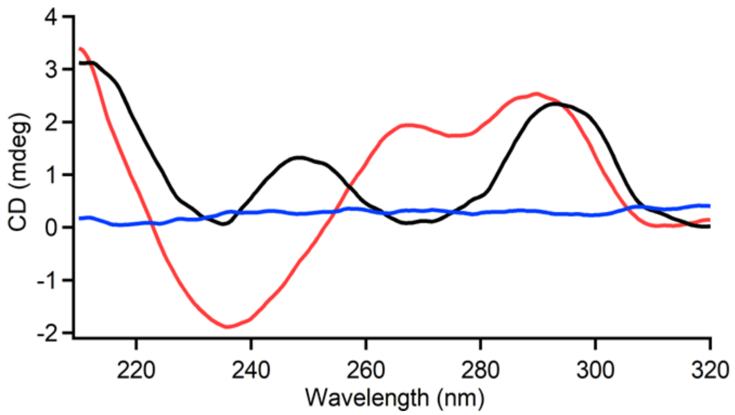

Supplementary Figure 2. Circular Dichroism spectra of a human telomeric G-quadruplex-forming sequence in sodium ions (black), and potassium ions (red) containing buffer (10 mM Tris, pH 7.4). Spectral curves were smoothed using the Savitzky-Golay algorithm with a 21-point sliding window.

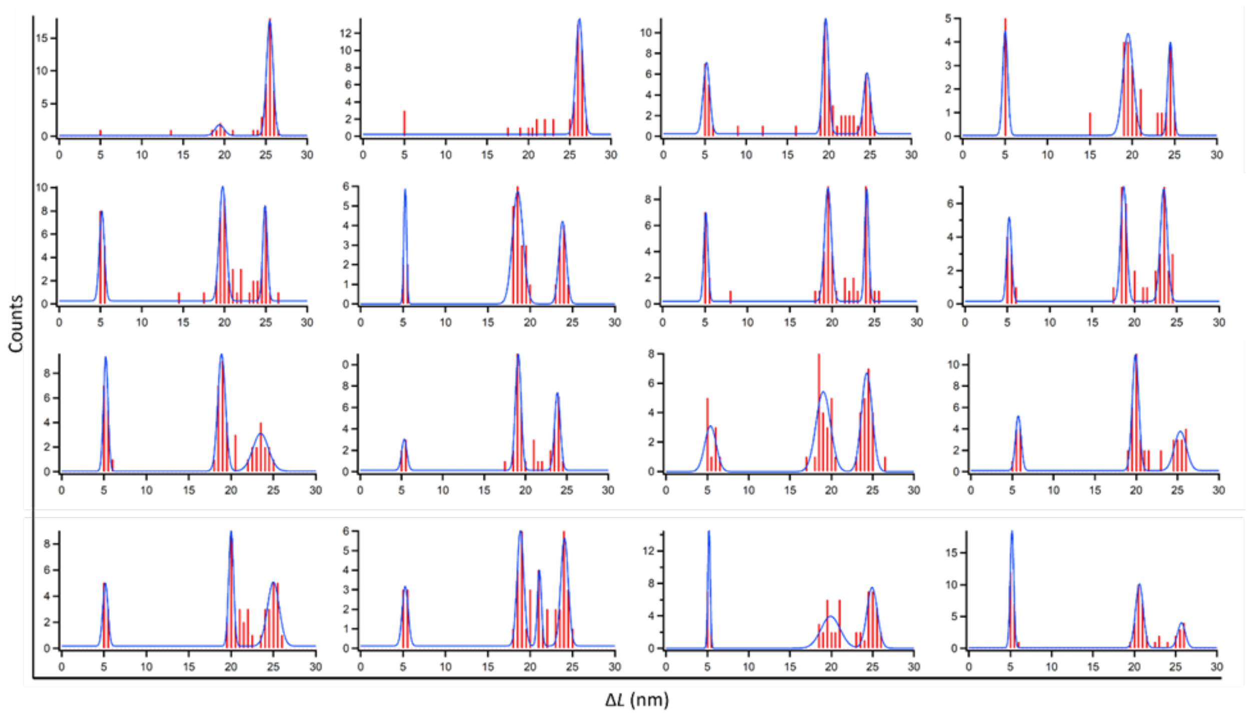

Supplementary Figure 3. Histograms show the distribution of the change-in-length (ΔL) measured for the G-quadruplex construct with one weak shearing handle in sodium buffer. Blue lines depict Gaussian multi-peak fits. The presence of three peaks indicate the following putative states of the target sequence: left peak, unfolding of the G-quadruplex, middle peak, DNC unlooping for the unfolded G-quadruplex, and right peak, DNC unlooping for the folded G-quadruplex.

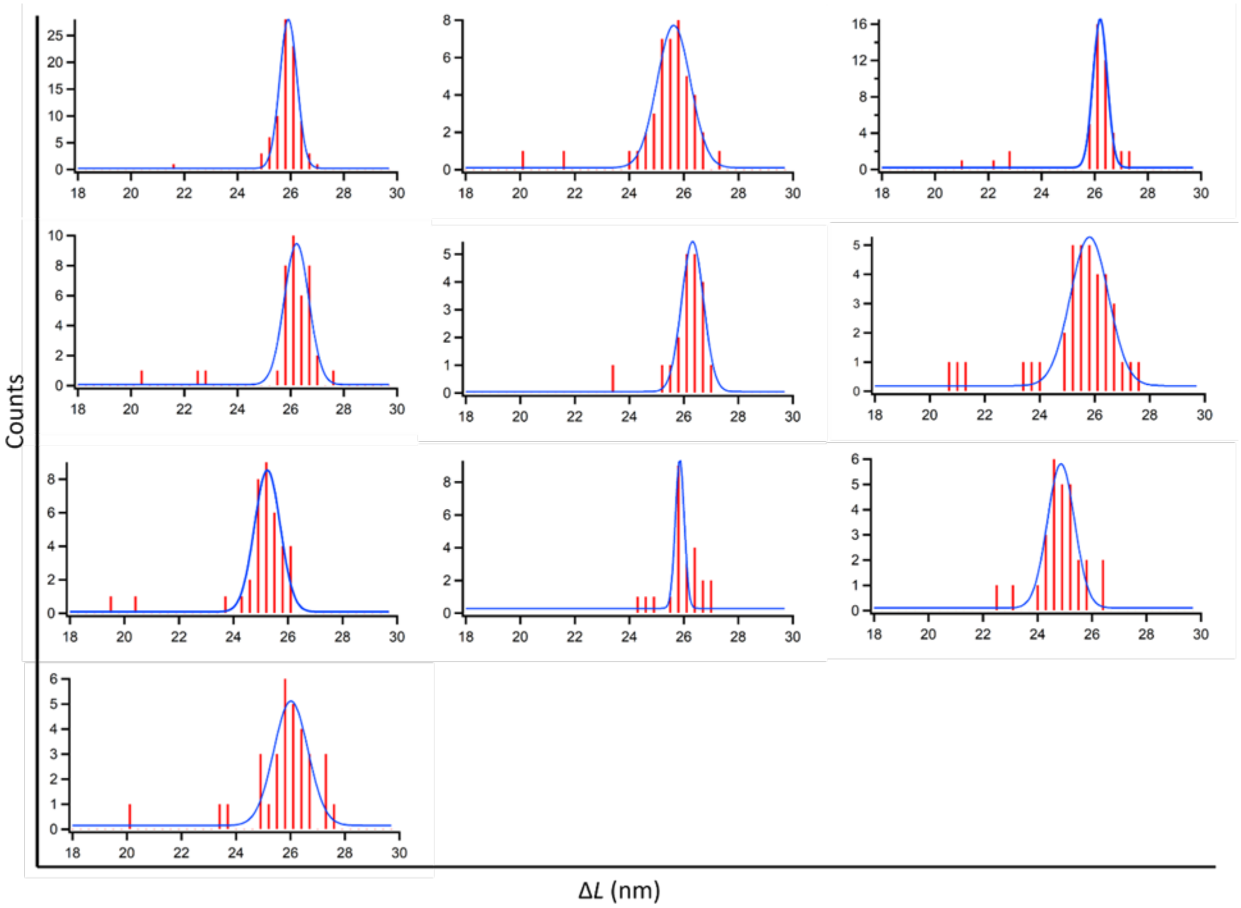

Supplementary Figure 4. Histograms show the distribution of the change-in-length (ΔL) measured for the G-quadruplex construct with one weak shearing handle in potassium buffer. Blue lines depict Gaussian peak fits. Each histogram exhibits a single predominant peak, putatively representing unlooping of the DNC.

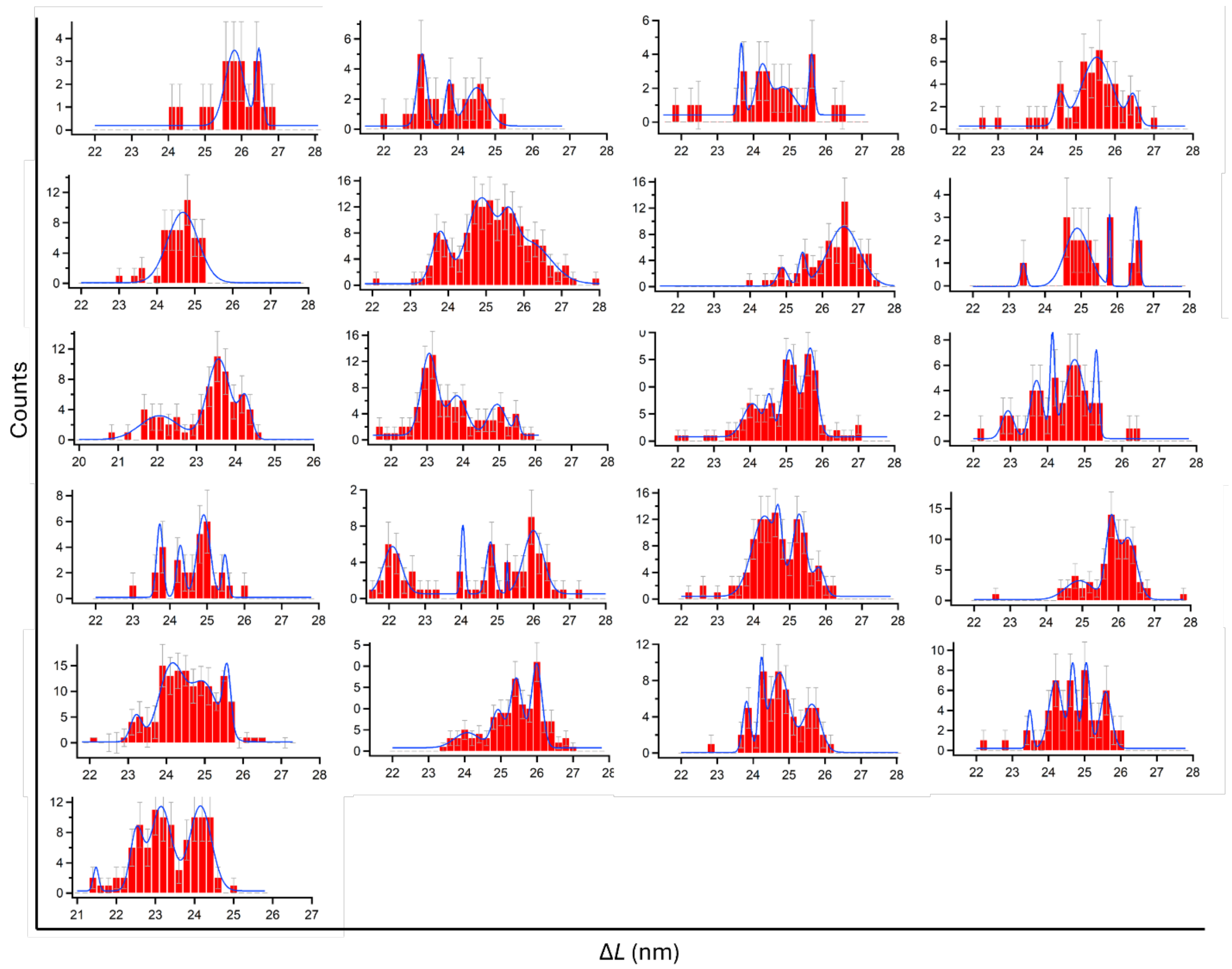

Supplementary Figure 5. Histograms show the distribution of the change-in-length (ΔL) measured for G-quadruplex constructs with multiple shearing handles in potassium buffer. Blue lines depict Gaussian multi-peak fits. The presence of several peaks putatively represents DNC unlooping events for different shearing handles. Error bars represent the standard deviation, calculated as the square root of the number of counts in each bin.

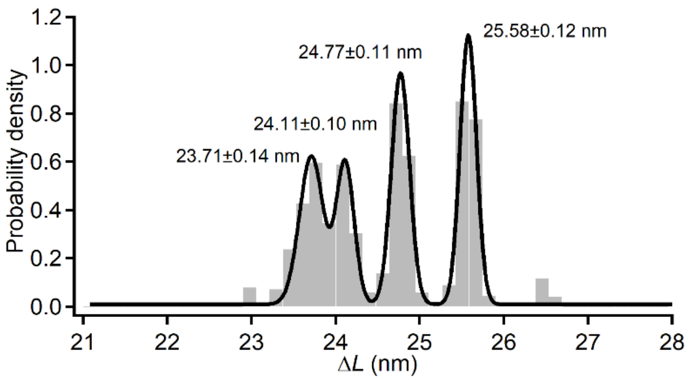

Supplementary Figure 6. Histogram of bootstrapped resampled data showing the distribution of the change-in-length (ΔL) measured using DNC. Solid black lines depict Gaussian multi-peak fits.

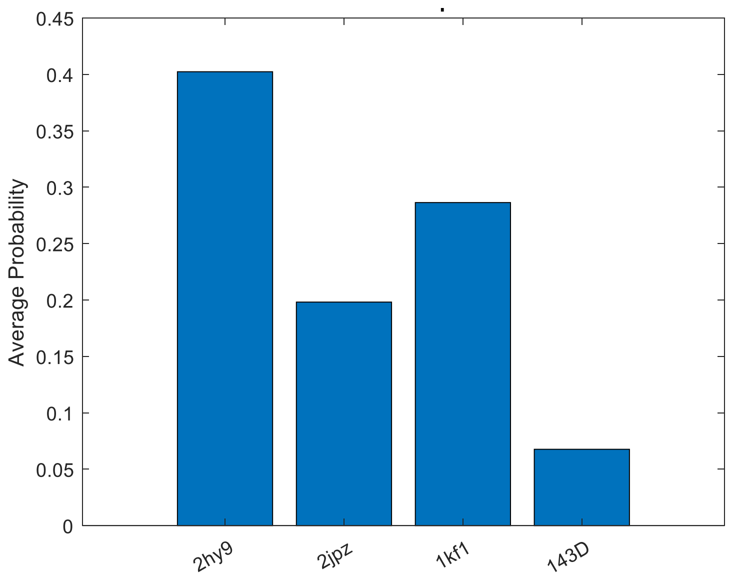

Supplementary Figure 7. Bar graphs showing the average estimated probabilities of candidate G-quadruplex conformations. Measurement distributions for the four possible conformations were modeled using Gaussian mixture models, with Gaussians centered at the expected mean positions of the four measurement sites, standard deviations fixed at 0.5 nm, and uniform weights. For each set of single-molecule caliper measurements, likelihoods were calculated for each conformation and then normalized across the four models. These normalized likelihoods were then averaged over all 21 molecules measured, with the resulting mean probabilities shown as bar plots.

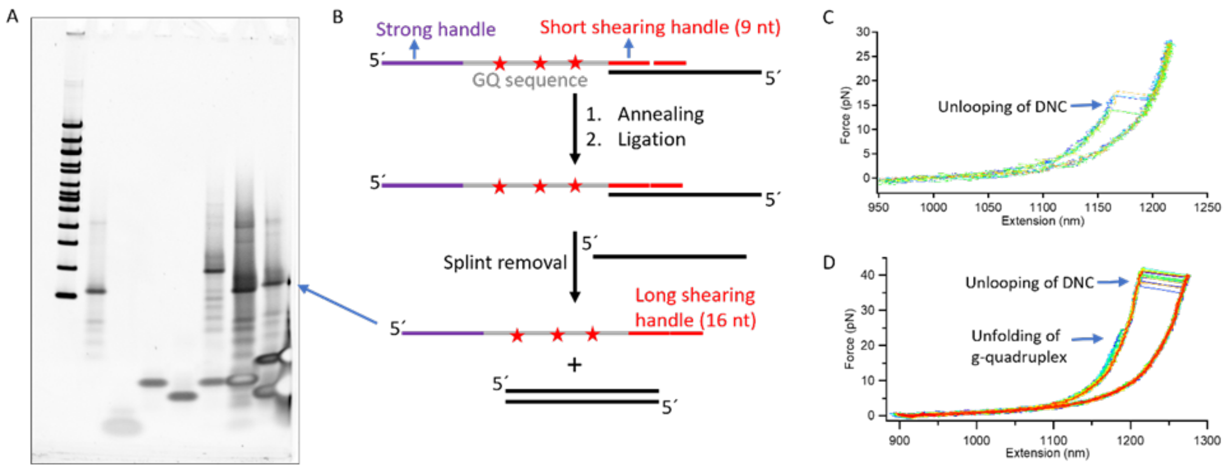

Supplementary Figure 8. Synthesis of g-quadruplex oligo with a longer shearing handle from the shorter shearing handle using DNA splint ligation. A) PAGE analysis (4-20% TBE gel, ThermoFisher Scientific) shows a successful synthesis of a target G-quadruplex with a long shearing handle (16 nt) from that of the short shearing handle (9 nt). Lanes from left to right: Lane 1, 50 bp ladder; Lane 2, target G-quadruplex strand with short (9 nt) shearing handle; Lane 3, handle extending oligo; Lane 4, long splint oligo; Lane 5, long splint oligo with its complementary oligo; Lane 6: annealed mixture; Lane 7: ligated mixture; Lane 8, long splint oligo removed mixture. The desired band (indicated by blue arrow) was purified. B) Schematic representation of the strategy for DNA splint ligation. The long blue arrow indicates the band of the desired product in (A) that was gel-purified to use in DNC measurements. Red stars indicate the position of incorporated azide in a loop nucleotide to label longer shearing handles by using DBCO-azide click chemistry as shown in Supplementary Figure 1. C) Force *vs.* extension graph of DNC with target G-quadruplex with the short shearing handle that confirms the unlooping of DNC at a lower force than the unfolding of G-quadruplex. D) Force *vs.* extension graph of DNC with the target G-quadruplex with a long shearing handle confirms the unlooping of DNC at a higher force than the unfolding of the G-quadruplex.

Supplementary Table 2. 5´ to 3´ end-to-end distances measured in different types of DNA G-quadruplex conformations reported by NMR[1–5].

**G-quadruplex conformations**	**Parallel** (PDB 1KF1)	**Antiparallel** (PDB 143D)	**Hybrid-1** (PDB 2HY9)	**Hybrid-2** (PDB 2JPZ)
5´-3´ (nm)	1.04	2.08	0.92	1.04

Supplementary Table 3. Distances between the 5´ end and the labeled residues measured in the different conformations of DNA g-quadruplex reported by NMR and X-ray crystallography[1–5].

**Distances**	**Parallel** (PDB 1KF1)	**Antiparallel** (PDB 143D)	**Hybrid-1** (PDB 2HY9)	**Hybrid-2** (PDB 2JPZ)
5´-5´ loop (nm)	1.89	1.87	1.07	1.79
5´- mid loop (nm)	2.67	1.77	2.36	1.81
5´-3´ loop (nm)	2.20	1.27	1.98	2.17
5´-3´ (nm)	1.04	2.08	0.92	1.04

Supplementary Table 4. Theoretical estimation of the change-in-distance due to unfolding of g-quadruplex based on the anti-parallel conformation (PDB 143D)[1] in sodium ion as previously determined and unlooping of DNC.

**Unfolding directions**	**End-to-end distance measured in a folded conformation (NMR) (nm)**	**G-quadruplex ΔL_{unfold} (nm)**	**DNC ΔL_{unloop} (nm)**
5´ - 5´ loop	1.87	-0.07	68.99
5´ - mid loop	1.77	2.28	65.81
5´- 3´ loop	1.27	5.48	62.63
5´- 3´	2.08	7.38	59.89

Supplementary references

(1) Wang, Y.; Patel, D. J. Solution Structure of the Human Telomeric Repeat d[AG3(T2AG3)3] G-Tetraplex. *Structure* **1993**, *1* (4), 263–282. https://doi.org/10.1016/0969-2126(93)90015-9.

(2) Lim, K. W.; Ng, V. C. M.; Martín-Pintado, N.; Heddi, B.; Phan, A. T. Structure of the Human Telomere in Na+ Solution: An Antiparallel (2+2) G-Quadruplex Scaffold Reveals Additional Diversity. *Nucleic Acids Res* **2013**, *41* (22), 10556–10562. https://doi.org/10.1093/nar/gkt771.

(3) Luu, K. N.; Phan, A. T.; Kuryavyi, V.; Lacroix, L.; Patel, D. J. Structure of the Human Telomere in K+ Solution: An Intramolecular (3 + 1) G-Quadruplex Scaffold. *J. Am. Chem. Soc.* **2006**, *128* (30), 9963–9970. https://doi.org/10.1021/ja062791w.

(4) Dai, J.; Carver, M.; Punchihewa, C.; Jones, R. A.; Yang, D. Structure of the Hybrid-2 Type Intramolecular Human Telomeric G-Quadruplex in K+ Solution: Insights into Structure Polymorphism of the Human Telomeric Sequence. *Nucleic Acids Research* **2007**, *35* (15), 4927–4940. https://doi.org/10.1093/nar/gkm522.

(5) Parkinson, G. N.; Lee, M. P. H.; Neidle, S. Crystal Structure of Parallel Quadruplexes from Human Telomeric DNA. *Nature* **2002**, *417* (6891), 876–880. https://doi.org/10.1038/nature755.
